# An updated review on the efficacy of adjuvant endocrine therapies in hormone receptor– positive early breast cancer

**DOI:** 10.3747/co.v16i0.455

**Published:** 2009-07

**Authors:** S. Verma, S. Sehdev, A. Joy, Y. Madarnas, J. Younus, J. A. Roy

**Affiliations:** * Odette Cancer Centre, Sunnybrook Health Sciences Centre, Toronto, ON; † The Oncology Group, William Osler Health Centre, Brampton, ON; ‡ Cross Cancer Centre, Edmonton, AB; § Kingston Regional Cancer Centre, Kingston, ON; || London Regional Cancer Center, London, ON; # L’Hôpital du Sacré-Coeur de Montréal, Montreal, QC

**Keywords:** Adjuvant endocrine therapy, hormonal therapy, aromatase inhibitors, early-stage breast cancer

## Abstract

The third-generation aromatase inhibitors (AIS) are largely replacing tamoxifen in the adjuvant treatment of early-stage breast cancer in postmenopausal women with hormone receptor–positive tumours. To date, multiple trials have been conducted comparing tamoxifen treatment with an AI, and all have demonstrated improved disease-free survival with AI treatment. Trials have included direct 5-year comparisons between tamoxifen and an AI, switching to an AI within 5 years after initial tamoxifen treatment, or extending treatment with an AI after 5 years of completed tamoxifen treatment. Some of these trials have been completed; others are ongoing; and head-to-head trial comparisons of individual AIS are also in progress. The present article summarizes the data obtained from various clinical trials of hormonal therapy for early breast cancer. It also reviews recent data so as to shed light on the current status of these therapies. The focus is on the efficacy of treatment with an AI. Toxicity is discussed in the second article in this supplement.

## 1. INTRODUCTION

The efficacy of tamoxifen as adjuvant therapy for women with estrogen receptor (ER)–positive early breast cancer has been clearly demonstrated. Adjuvant tamoxifen treatment has been associated with a 31% reduction in the annual breast cancer mortality rate among hormone receptor–positive women with early breast cancer 1, initially making it a standard of care for this patient population. However, with the advent of the aromatase inhibitors (AIS)—namely anastrozole, exemestane, and letrozole—the standard of care has been evolving. The efficacy of the AIS in comparison with tamoxifen has been examined in recently completed or ongoing trials, and although data from these trials have shown a definitive benefit over tamoxifen in terms of disease-free survival (DFS), benefit in terms of overall survival (OS) is less clear. Early trials of tamoxifen often used OS as the primary endpoint, but recent trials comparing the AIS with tamoxifen have used other endpoints in addition to OS to demonstrate clinical benefit, and so it is important to review the endpoints in the context of current clinical care of postmenopausal women with breast cancer.

## 2. TRIAL ENDPOINTS

### 2.1 What Are the Clinically Meaningful Endpoints in Trials of Adjuvant Endocrine Therapy?

Clinically meaningful endpoints are distinct measurements or analyses that reflect how a patient feels, functions, or survives 2. Survival benefits are considered the ideal primary efficacy endpoints by the U.S. Food and Drug Administration (FDA) 3 and thus improvement in OS has long been considered the “gold standard” for evaluating the efficacy of cancer drugs in phase III clinical trials in the adjuvant setting.

Although OS is the most objective measure of efficacy, its evaluation requires prolonged follow-up, and consequently, the use of surrogate endpoints has become acceptable. In addition, OS measurements are affected by the development of effective salvage treatments for patients with metastatic disease, and they therefore may not be a pure efficacy endpoint in the evaluation of adjuvant therapy. The most widely used surrogate endpoints in oncology are DFS or event-free survival, the latter being the time from the beginning of treatment until a patient experiences a recurrence, a new primary cancer, or death. For some cancers, DFS is now recognized as an acceptable endpoint for evaluating therapies 4. Another endpoint whose importance is also recognized is relapse-free survival or recurrence-free survival (RFS), which is the time from the start of treatment until a recurrence of the original cancer. Time to distant recurrence (TTDR), which, as the term implies, does not include local or regional recurrence, therefore depends on distant events. With breast cancer, distant events in patients on adjuvant therapy have been observed to be more frequent within the first 2–3 years following surgery and to be associated with poorer survival 5.

Early trials of tamoxifen for the adjuvant treatment of breast cancer used OS and DFS as primary efficacy endpoints. However, these early trials were comparing tamoxifen with placebo, rather than with an active drug. The more recent AI trials have used DFS as the primary endpoint, with TTDR and RFS as surrogate endpoints of clinical benefit (Table I). Although very early trials of tamoxifen did not include contralateral breast cancer (CLBC) in estimations of DFS 7, CLBC is now commonly accepted and taken into account when evaluating DFS as a primary endpoint. However, confusion arises from the fact that the definition of CLBC in itself varies between studies, with some studies including ductal and lobular cancer *in situ* (DCIS and LCIS) within CLBC and others not 14. Furthermore, evaluation of second primary cancers may include or exclude CLBC. As well, in some recent studies, secondary endpoints such as time to recurrence (TTR) and RFS have varied with respect to inclusion of CLBC. For example, in the Arimidex, Tamoxifen, Alone or in Combination (ATAC) study comparing anastrozole with tamoxifen 15, TTR included new contralateral tumours; but in the Breast International Group (BIG) 1–98 study comparing letrozole with tamoxifen 13, RFS did not include CLBC. These inconsistencies in trial methodology can make a comparison of data from different trials difficult, particularly if the absolute benefit is small.

### 2.2 How Does DFS Correlate with OS?

The use of DFS as a surrogate endpoint for OS is most appropriate in settings in which recurrence of the disease is responsible for a major component of mortality in the treated population, which is the case for most solid tumours 16. According to the 2007 FDA guidance document on clinical trial endpoints for the approval of cancer drugs and biologics, “DFS can be an important endpoint in situations where survival may be prolonged, making a survival endpoint impractical” 3. The primary basis of FDA approval for adjuvant hormonal and cytotoxic therapies for breast cancer and for adjuvant therapy for colon cancer has been DFS.

With respect to early breast cancer, OS alone was often used as the primary endpoint in early trials of adjuvant therapy. However, in interim analyses of some studies, it became evident that OS benefit would not be apparent and that other measures such as DFS also had to be considered. For example, in the National Surgical Adjuvant Breast and Bowel Project (NSABP) B-14 randomized trial involving 2644 ER-positive node-negative women, no significant advantage in OS was found after 4 years of follow-up (93% and 92% for tamoxifen and placebo respectively, *p* = 0.3); however, DFS was 83% and 77% respectively (*p* < 0.00001) in the two cohorts 6. Consequently, DFS was used as an endpoint and has been found to be an adequate surrogate for OS. In a 2007 meta-analysis of 10 randomized clinical trials of AIS versus tamoxifen, a strong correlation between DFS and OS was found 17, supporting the use of DFS as a surrogate endpoint for OS in breast cancer adjuvant endocrine trials. Further support was demonstrated in a recent analysis of 128 breast cancer adjuvant trials in which the difference in 2-year DFS was found to be a significant predictor of the difference in 5-year OS 18.

It is also becoming evident that, as the length of follow-up increases, competing causes of death confound survival findings. Recent data on node-negative ER-positive early breast cancer treated with adjuvant tamoxifen at a median follow-up of 64 months showed that affected women had a considerably higher risk of dying from causes unrelated to breast cancer (10-year probability of all-cause mortality was 24% and of breast cancer mortality was 4%) 19. In the 100-month analysis of the ATAC trial, a difference in OS and an increasing number of deaths from other causes were noted in both groups 15,20, perhaps associated with the fact that, on additional follow-up, the average age of the patients enrolled in the trial is now 72 years. On the other hand, significant advantages in DFS, CLBC, and TTR were observed in the anastrozole group, as was a 16% lower risk of distant recurrence 20. Similarly, at a 51-month evaluation of letrozole versus tamoxifen, patients randomly assigned to letrozole were 18% less likely to have a DFS event and were also significantly less likely to experience distant metastasis 21. Thus, although OS is the ideal standard endpoint that fully reflects the effects of a particular therapy, DFS is not only an acceptable substitute, but also allows for a more efficient means to evaluate efficacy. However, as the FDA guidance document recognizes, critical issues include the adequacy of the duration of study follow-up to evaluate the durability of a DFS benefit and the variety of definitions for DFS. In most breast cancer adjuvant trials, DFS typically includes local, regional, or distant recurrence, and death from any cause. However, some studies include DCIS and LCIS within the definition of DFS, and others do not (Table II). Consequently, a panel of experts in breast cancer clinical trials proposed a standardized definition (STEEP), adopting the more precise term “invasive disease-free survival,” which would include ipsilateral invasive breast tumour recurrence, regional invasive breast cancer recurrence, distant recurrence, death from any cause, CLBC (invasive), and second primary non-breast invasive cancer 14. Indeed, breast cancer-related death may be a more meaningful marker than death from any cause or from second primary non-breast cancers, and should potentially replace these endpoints in the definition of DFS.

## 3. BASIS OF ENDOCRINE AND ADJUVANT ENDOCRINE THERAPY TRIALS IN HORMONE RECEPTOR–POSITIVE BREAST CANCER

Women with breast cancer may have tumours with receptors for hormones such as estrogen or progesterone. Circulating estrogen binds to its cognate receptor in women with ER-positive breast tumours, which induces cell proliferation 23; consequently, prevention of tumour growth is based on blocking this interaction. A woman’s ovaries produce estrogen until menopause, after which smaller amounts are produced in peripheral tissues through conversion of testosterone and androgen precursors (androstenedione and dehydroandrosterone) produced by the adrenal gland 24. This conversion occurs through the action of the cytochrome P450 enzyme, aromatase, or CYP19 25. Tamoxifen acts by blocking estrogen receptors on tumour cells, but the AIS block the ability of the aromatase enzyme to produce estrogen 26. Anastrozole and letrozole are nonsteroidal agents that bind reversibly to the aromatase enzyme; exemestane is a steroidal agent that binds irreversibly to aromatase 25. Both types of AI reduce estrogen to less than 10% of the level before treatment commenced 25. Because of their profound estrogen depletion effect, the AIS are increasingly being shown to be more effective in reducing recurrences, and because they do not have the estrogen agonist properties of tamoxifen on uterine tissue and the coagulation cascade, they have a different and better safety profile than that of tamoxifen. Current data support the use of AIS in the adjuvant setting, and treatment guidelines and consensus documents are evolving toward incorporation of AIS for the treatment of early-stage breast cancer 27–30.

The risk of breast cancer recurrence is highest within the first 5 years of primary chemotherapy 31, and even with tamoxifen treatment, 50% of women who experience a recurrence do so within the first 5 years following surgery 1. Furthermore, recurrence—in particular, distant recurrence—is associated with increased mortality 32. In a retrospective study of 1616 patients with early breast cancer, the survival probability for women with distant recurrence was 41% compared with 92% for those with no recurrence 32. Therefore, reducing the risk of recurrence is of prime importance in the quest for improved survival. Trials with the AIS have shown lower rates of recurrence than are seen with tamoxifen, and the decline is evident within 2 years of therapy start 15.

## 4. UPDATES FROM ADJUVANT ENDOCRINE THERAPY TRIALS

Several trials with AIS for adjuvant endocrine treatment in postmenopausal women with early-stage breast cancer are either underway or have been completed. In addition to trials for upfront AI treatment, there have also been trials to evaluate switching from tamoxifen to an AI, or extending treatment with an AI after tamoxifen for 5 years (see Buzdar *et al.* 33 for a recent review). The reported DFS benefit in these trials is shown in Table III.

### 4.1 Upfront Trials

A recent meta-analysis carried out by the Early Breast Cancer Trialists Group (EBCTG) of a number of AI trials showed that, with AIS, breast cancer recurrences are significantly less frequent than they are with tamoxifen 40. The analysis focused on ER-positive women, and the endpoints were all breast cancer recurrences, including local, distant, and contralateral, and death. At 5 years, AI therapy was associated with an absolute 2.7% reduction in breast cancer recurrence and an absolute 1.6% decrease in breast cancer mortality 40. The two major upfront trials carried out so far were the ATAC trial, a phase III double-blind trial comparing anastrozole and tamoxifen either alone or in combination for more than 5 years 9,41, and the BIG 1–98/International Breast Cancer Study Group (IBCSG) 18–98 study, a double-blind phase III trial comparing letrozole or tamoxifen alone or tamoxifen followed by letrozole and vice versa 13.

The ATAC trial enrolled 9366 women from 21 countries, including 635 from Canada, all of whom were postmenopausal with early-stage breast cancer 9,41,42, and all of whom had completed surgery, plus radiation and chemotherapy (where given). The women were randomly assigned to receive either 1 mg anastrozole alone (*n* = 3125), 20 mg tamoxifen alone (*n* = 3116), or a combination of the two (*n* = 3125). After the first analysis at 33 months, the combination arm was dropped when no benefit was demonstrated as compared with tamoxifen alone 43. After a follow-up of 68 months, women with hormone receptor–positive disease on anastrozole showed an improvement in DFS [hazard ratio (HR): 0.83; 95% confidence interval (CI): 0.73 to 0.94; *p* = 0.005], an improvement in TTR (HR: 0.74; 95% CI: 0.64 to 0.87; *p* = 0.002), and a reduced occurrence of CLBC (53% reduction; 95% CI: 25% to 71%; *p* = 0.001) as compared with women on tamoxifen 41. Further, fewer serious side effects such as endometrial cancer, thromboembolic events, vaginal bleeding, and hot flushes were observed. Bone fractures and joint pain were more common in the anastrozole group, although the incidence of hip fracture was low and similar in both groups 41.

Follow-up of the ATAC trial beyond 5 years of treatment and data obtained at 100 months showed significant improvement in DFS, TTR, TTDR, and CLBC in women on anastrozole, although no difference in OS was noted 14. The lower recurrence rate with anastrozole was maintained after treatment was completed, in particular for the hormone receptor–positive group, in whom the absolute benefit of 2.8% at 5 years increased to 4.8% at 9 years (*p* = 0.0001). That finding suggests a carryover effect for anastrozole, whereby women continue to benefit from treatment with anastrozole after stopping treatment. Although fracture rates overall were higher in women on anastrozole during the treatment period, the incidence was the same for both groups after treatment had been completed 15. These long-term data from the ATAC trial indicate a continued benefit with anastrozole even after treatment has been terminated, without further treatment-related side effects.

The BIG 1–98/IBCSG 18–98 study enrolled 8010 hormone receptor–positive postmenopausal women worldwide, including 20 from Canada 13,42. Women were randomly assigned to 2.5 mg letrozole or 20 mg tamoxifen alone (1835 patients) or to tamoxifen for 2 years followed by letrozole and vice versa (6193 patients). The latter two arms were designed to compare the efficacy of a sequence strategy with that of an upfront AI strategy. At 25 months, women on letrozole showed an improvement in DFS (8.8% vs. 10.7%; *p* = 0.003), defined as the time from randomization to the first local, regional, or distant recurrence; a new invasive cancer in the contralateral breast; a second, non-breast cancer; or death without a preceding cancer event 13. At a median follow-up of 51 months, three additional endpoints were included in the analysis, namely DFS excluding second non-breast cancers; TTR defined as DFS, but excluding second, non-breast cancers; and exclusion of data from patients who died without a recurrence of breast cancer. Women randomly assigned to continuous therapy on letrozole showed an 18% reduction in the risk of an event (*p* = 0.007), and the 5-year DFS estimates for letrozole and tamoxifen were 84% and 81% respectively. A CLBC occurred in 0.6% of women on letrozole as compared with 1.1% of women on tamoxifen, and a 1.2% absolute decrease in distant recurrence was observed among women on letrozole. Fewer gynecologic and thromboembolic events, but more bone fractures, arthralgia, and low-grade hypercholesterolemia were experienced by women on letrozole as compared with those on tamoxifen 44.

Updated results at a median follow-up of 76 months (reported at the 2008 San Antonio Breast Cancer Symposium) continued to support improved survival for patients treated with letrozole 45; however, the weight of evidence favouring an upfront AI over tamoxifen resulted in the unblinding of the tamoxifen arm shortly after the 51-month analysis, and 619 patients (25.2%) selectively crossed over to letrozole, mostly in years 3–5. The unblinding confounded the statistical analysis, and the 76-month comparison of upfront letrozole with tamoxifen has been difficult; however, letrozole has continued to show superior efficacy over tamoxifen (Mouridsen HT. Letrozole monotherapy vs. tamoxifen monotherapy or vs. letrozole in sequence with tamoxifen for post-menopausal women with endocrine-responsive early breast cancer. Presented at the San Antonio Breast Cancer Symposium 2008; San Antonio, TX, U.S.A.; December 10–14, 2008). A trend toward an OS benefit is also now evident (*p* = 0.08); however, whether this trend is real or confounded by the crossover of patients from tamoxifen to letrozole is difficult to assess 46.

The Tamoxifen Exemestane Adjuvant Multinational (TEAM) study was initiated in 2001 in 9 countries to examine the efficacy of exemestane versus tamoxifen in 9775 hormone-sensitive women with early breast cancer 46. However, the study design was revised in 2004, and patients on tamoxifen were switched to exemestane after 2.5–3 years, when the Intergroup Exemestane Study (IES) study (described in the next subsection) reported superior results for a switch from tamoxifen to exemestane after 2–3 years. At 2.75 years, the event rate in both groups was low, but exemestane was associated with an improvement in DFS 46. Also, being that TEAM is an open-label trial, significant rates of discontinuation of the study drug have occurred (30% for tamoxifen, 19% for exemestane), which may confound study analysis in the future.

Two key head-to-head trials of AIS as adjuvant treatment for women with early breast cancer are underway. The Femara versus Anastrozole Clinical Evaluation (FACE) is a phase III, open-label, randomized, multicentre trial designed to test whether anastrozole or letrozole has superior efficacy as adjuvant treatment for 5 years in postmenopausal women with hormone receptor–positive, node-positive disease 47. The trial is recruiting 4000 patients from 250 international sites, and recurrence and survival will be assessed every 12 months. Another phase III trial, MA.27 from the National Cancer Institute of Canada (NCI C), is comparing anastrozole with exemestane in the treatment of postmenopausal women with hormone receptor-positive disease (visit www.clinicaltrials.gov/ct/show/NCT00066573 for details) and has completed accrual. Results of these trials should yield useful information about the individual effects and relative efficacy of the AIS.

### 4.2 Switch and Sequential Trials

The Italian Tamoxifen Arimidex (ITA) trial has been evaluating a switch to anastrozole after 2–3 years of tamoxifen 12,34. In that trial, 448 women with node-positive, ER-positive tumours were randomly assigned to anastrozole or continuation on tamoxifen after 2–3 years on tamoxifen 12. At 64 months, OS was superior in those who had switched to anastrozole, but the difference did not reach statistical significance 34. However, RFS was superior in the anastrozole group as compared with the group that remained on tamoxifen (*p* = 0.01). Gastrointestinal complaints, musculoskeletal disorders, fractures, disorders of lipid metabolism, and hyperglycemia were more common in women on anastrozole. Women on tamoxifen developed more venous disorders and gynecologic changes (including endometrial cancer). Notably, however, the trial was unable to fulfil the sample size calculations because of competing trials, and the results are therefore based on the patients who were able to be recruited to the trial.

Two additional trials, the Arimidex–Nolvadex (ARNO 95) study and the Austrian Breast and Colorectal Cancer Study Group (ABCSG) trial 8, which together involve 3224 women with ER-positive early-stage breast cancer, are also examining the effect of continuation on tamoxifen after 2 years, as compared with a switch to anastrozole for a further 3 years 22. At 28 months, fewer events (local or metastatic recurrence, contralateral breast cancer, or death) were noted in the anastrozole group than in the tamoxifen group (*p* ≥ 0.001), representing a 40% reduction in the risk of an event in women switched to anastrozole after being on tamoxifen for 2 years. Although more fractures and bone pain were recorded in the anastrozole group, fewer thromboses occurred 22. A German subgroup of the ARNO study, involving 979 patients, also reported improved OS and DFS at a median follow-up of 30 months, with fewer adverse events reported in the anastrozole group 37.

A meta-analysis of the three aforementioned trials confirmed that, among patients who switched to anastrozole as compared with those who remained on tamoxifen, fewer disease recurrences and deaths were observed, resulting in significant improvements in DFS (*p* < 0.0001), event-free survival (*p* < 0.0001), distant recurrence-free survival (*p* = 0.002), and OS (*p* = 0.04) 35.

In contrast with the switch trials, in which events are analyzed from the point of the switch (after 2–3 years of treatment with tamoxifen), sequential trial strategies analyze events from the start of treatment. Sequential and switch trials investigate the same intervention, but they are conducted in different patient groups and are therefore expected to provide different results. The ABCSG 8 trial was recently completed, and the sequencing strategy continued to show improved benefit as compared with tamoxifen alone for 5 years 48. However, it should be noted that the sample size and statistical calculations were completed after ARNO and ABCSG combined all their accrued patients. The results from ABCSG alone provide a clinical clue, but cannot truly be interpreted as a trial result.

The IES is a double-blind randomized trial that compares a switch to exemestane in postmenopausal women with early breast cancer who have been on tamoxifen for 2–3 years with continuation of tamoxifen alone for a total of 5 years 11. The trial enrolled 4742 women from 37 countries who had been on tamoxifen for 2–3 years and who were then randomly assigned to either switch to exemestane or continue on tamoxifen for up to 5 years 11. After a median follow up of 55.7 months, evaluation of the data showed a 24% risk reduction and a 3.3% absolute benefit of switching to exemestane. After treatment, exemestane maintained its DFS advantage, which is consistent with a carryover effect for the tamoxifen–exemestane switch strategy similar to the effect seen with tamoxifen alone. The differences in carryover effect seen in ATAC and IES may have occurred because, during the first 2–3 years of treatment, tamoxifen failed to eliminate micrometastases that later emerged as recurrences, or because patients in the IES trials received only 3 years of AI therapy as compared with 5 years of AI therapy in ATAC. Furthermore, although an OS benefit was seen, it appears that that benefit is driven by fewer noncancer- related deaths in the exemestane arm 49. An endometrial substudy determined that women who switched to exemestane had significant reductions in endometrial thickening and uterine volume as compared with women who remained on tamoxifen at 24 months post treatment 50.

The most recent analysis for the BIG 1–98/IBCSG 18–98 trial, in addition to reporting the monotherapy analysis with either letrozole or tamoxifen alone, also included an analysis on sequencing to letrozole for 3 years following 2 years on tamoxifen and vice versa 13. Approximately 1550 women were given letrozole followed by tamoxifen or vice versa. At a median follow-up of 76 months, sequential treatments did not improve DFS as compared with upfront letrozole alone 45. The study was designed to assess superiority of the sequence arms over letrozole monotherapy, and based on the results, all that can be said is that the sequence strategy is not superior to an upfront AI strategy.

The EBCTG recently carried out a meta-analysis of the ABCSG 8, ARNO 95, IES/BIG 2–97, and ITA trials and showed that AIS are associated with significantly fewer breast cancer recurrences, even in the switch setting, and furthermore, a statistically significant improvement in mortality rates 40. However, these findings should be interpreted with caution given the significant heterogeneity of study populations in the switch trials.

### 4.3 Endocrine Extension Trials

Several trials have examined the efficacy of adjuvant treatment extended beyond the standard 5-year duration. These have included extended treatment on tamoxifen or an AI beyond 5 years, or sequential treatment with an AI in women who were disease-free after 5 years on tamoxifen. The Adjuvant Tamoxifen, Longer Against Shorter (ATLAS) randomized trial is comparing 10 years with 5 years of tamoxifen in 11,500 women (59% ER-positive). A lower recurrence rate has been observed among women continuing on tamoxifen at a mean of 4.2 years following randomization 51. Similarly, the Adjuvant Tamoxifen—To Offer More (attom) trial has randomly assigned 6934 women in the United Kingdom who have already received 5 years of tamoxifen to cessation or continuation for another 5 years 52. With a median follow-up of 4.2 years, fewer recurrences (although not statistically significantly fewer: *p* = 0.4) were observed in the group on extended treatment, but the risk of endometrial cancer was also observed to be doubled. Of interest, an earlier study found no benefit for extended treatment with tamoxifen in women with node-negative cancer 53. In that study, DFS was 82% in patients who discontinued tamoxifen as compared with 78% (*p* = 0.07) in those who continued to receive it.

The double-blind MA.17 trial from the NCI C Clinical Trials Group is looking at the effect of either letrozole or placebo in women who have already undergone standard 5-year treatment with tamoxifen 10,54. Following approximately 5 years of tamoxifen treatment, MA.17 randomly assigned 5187 postmenopausal women with early-stage breast cancer to either letrozole or placebo. The 4-year DFS is 94.4% and 89.8% for patients receiving letrozole and placebo respectively, representing an absolute reduction of 4.6% for patients on letrozole (*p* < 0.001) 55. After a median follow-up of 30 months, the trial was unblinded, and patients who received placebo were offered letrozole; 66% of patients on placebo opted to take letrozole 56. At 2.8 years from unblinding, 2% in the letrozole group as compared with 4.9% in the placebo group experienced an event 57. These data indicate that letrozole improves DFS and distant DFS, even when a substantial period of time has elapsed since the discontinuation of prior adjuvant tamoxifen.

The ABCSG trial 6a is similar to MA.17: It is examining the efficacy of 3 years of anastrozole treatment in women who have completed 5 years of tamoxifen treatment, with or without the first-generation AI aminoglutethimide for the first 2 years of therapy 39. Disease-free patients (*n* = 856) were randomly assigned to receive either 3 years of anastrozole or no further treatment. At a median follow-up of 62.3 months, women who received anastrozole (*n* = 387) had a statistically significantly reduced risk of recurrence (locoregional recurrence, CLBC, or distant metastasis) as compared with women who received no further treatment (*p* = 0.031) 39.

The NSABP B-33 trial was designed to examine whether exemestane treatment (versus placebo) prolongs DFS following 5 years of tamoxifen treatment 58. However, following the results of the MA.17 extended trial with letrozole, NSABP B-33 was unblinded after 29 months, and women on placebo were offered exemestane. At 30 months’ median follow-up, women originally assigned to exemestane showed an improvement in 4-year DFS and RFS^58^.

### 4.4 Trials in Premenopausal Women

The addition of 5 years of tamoxifen in premenopausal women with hormone receptor–positive disease is associated with a 40% reduction in recurrence 1,8. More recently, drug-induced ovarian suppression has been examined. The Zoladex Early Breast Cancer Research Association trial showed that goserelin treatment was as effective as cyclophosphamide, methotrexate, and fluorouracil chemotherapy for treatment of pre- and perimenopausal women with node-positive, ER-positive breast cancer 59. In the Zoladex in Premenopausal Patients trial, premenopausal women were randomly assigned to goserelin alone for 2 years, tamoxifen alone for 2 years, goserelin plus tamoxifen for 2 years, or no endocrine treatment, after surgery and standard chemotherapy or radiotherapy, or both 60. Goserelin either alone or with tamoxifen was associated with a significant decrease in the risk of recurrence.

Treatment with AI alone has not been recommended in premenopausal women with functioning ovaries following chemotherapy. In these women, though AIs may reduce estrogen production in peripheral tissue to some extent, ovarian estrogen production is maintained or increased, which may lead to unfavourable effects on breast cancer risk of recurrence 61. However, AI treatment together with ovarian suppression is currently being evaluated for premenopausal patients with hormone-responsive early-stage breast cancer. The ABCSG-12 trial is examining the efficacy of anastrozole or tamoxifen in combination with goserelin, with or without zoledronic acid, in premenopausal women with hormone-responsive early breast cancer 62, and at a median follow-up of 60 months, no significant difference in DFS between the two endocrine therapies has been observed. However, the addition of zoledronic acid to endocrine therapy resulted in a 36% improvement in DFS (*p* = 0.01) at a median follow-up of 48 months, and fewer patients on that combination experienced bone metastases 63. Further follow-up from that study, and from other similar trials [AZURE (neoadjuvant zoledronic acid to reduce recurrence), for example], is eagerly anticipated to help clarify the role of adjuvant bisphosphonates for patients with early-stage breast cancer.

The IBCSG is currently conducting three trials in European and North American premenopausal women 64,65. In the ongoing Suppression of Ovarian Function trial, women with endocrine-responsive disease who remain premenopausal after surgery or after the completion of chemotherapy are randomly assigned to tamoxifen alone for 5 years as compared with ovarian function suppression (triptorelin or oophorectomy) in combination with either tamoxifen or exemestane. The Tamoxifen and Exemestane study is enrolling premenopausal women on triptorelin and either tamoxifen or exemestane from the start of their adjuvant therapy. The Premenopausal Endocrine Responsive Chemotherapy trial is randomly assigning women to ovarian suppression plus either tamoxifen or exemestane, or ovarian suppression plus either tamoxifen or exemestane plus chemotherapy. Trials such as these should yield important information concerning the efficacy of AIs together with ovarian suppression as adjuvant treatment in premenopausal women.

## 5. OPTIMAL DURATION OF AI TREATMENT

Several trials are being conducted to determine the optimal duration of AI treatment for postmenopausal women with early breast cancer. The MA.17R trial was initiated in 2004 (visit cancer.gov/clinicaltrials/CAN-NCIC-MA17R for details) to compare DFS in subjects who receive 5 years of letrozole or placebo after having received approximately 5 years (4.5–6 years) of aromatase inhibitor therapy (letrozole, anastrozole, or exemestane), including those who have received 5 years of adjuvant letrozole as part of the MA.17 trial. In the ABCSG-16 Secondary Adjuvant Long-Term Study in the Arimidex trial, women who have been free of recurrence after approximately 5 years of endocrine therapy will receive either 2 years or 5 years of extended adjuvant therapy with anastrozole 66. The NSABP B-42 trial will determine whether prolonged adjuvant hormonal therapy with letrozole will improve DFS in postmenopausal women with hormone receptor– positive breast cancer who have completed 5 years of therapy with an AI or 5 years of therapy with a combination of up to 3 years of tamoxifen followed by an AI 67.

With ongoing studies looking into the benefits of an additional 5 years of treatment with an AI, some physicians question whether patients will be willing to take these medications for this length of time. To address that concern, a survey was recently conducted among patients with early breast cancer and the physicians who treat them. Physicians were asked to indicate the minimum incremental benefit that they believed would justify 5 additional years of AI therapy. Most indicated that they would prescribe an AI for a further 5 years only if it produced an additional 1%–2% benefit in OS. When patients with early breast cancer on an AI therapy were asked the same question, one third responded that they would continue AI therapy for a further 5 years even if it offered a less-than-1% incremental benefit in OS. These results suggest that physicians may be more risk-averse than are their patients; the results are also encouraging in that they show that most patients tolerate their AI medication well enough to consider a further 5 years of therapy for a very small potential benefit 68.

## 6. RESISTANCE TO ENDOCRINE THERAPY AND FACTORS AFFECTING DRUG ACTION

Because adjuvant endocrine therapy is generally a long-term treatment, the development of resistance and other issues affecting drug efficacy must be considered because these events will have an effect on the benefits of treatment. Drug resistance may be intrinsic or acquired. Intrinsic (*de novo*) resistance refers to a lack of response at initial exposure to the drug; acquired resistance develops during therapy in patients who are initially responsive. Host factors, treatment type, and the biology of the tumour can all have a role to play in the development of resistance.

### 6.1 Host Factors

Tamoxifen is converted to anti-estrogenic metabolites that are thought to be more potent than tamoxifen itself. One of the active metabolites, endoxifen, is produced through the action of the cytochrome P450 enzyme CYP2D6, and women who have particular allelic variants of the genes encoding this enzyme are poor metabolizers of tamoxifen 69,70. In family studies, the poor-metabolizer phenotype behaves as an autosomal recessive trait with an incidence of between 5% and 10% in the white population of Europe and North America, and leads to deficient metabolism of more than 20 commonly prescribed drugs 71. In contrast, certain populations have multiple copies of the *CYP2D6* gene and are consequently good metabolizers. For example, 29% and 21% respectively of Ethiopians and Saudi Arabians were found to carry extra *CYP2D6* genes, whereas 1%–2% of Swedish, German, Chinese, and black Zimbabwean populations had multiple copies 72,73. Poor metabolizers of tamoxifen (those with *CYP2D6* alleles *4, *5, *10, *41) are at increased risk of recurrence, while those with high-activity variants have a more favourable outcome 74. However, high metabolizers experience more side effects and are more likely to discontinue treatment 75. Thus, paradoxically, the patients who are most likely to benefit from tamoxifen treatment are also the most likely to experience adverse effects and to discontinue treatment. In addition, there is also a suggestion that the HER2/*neu* oncogene encodes a transmembrane tyrosine kinase receptor with extensive homology to the epidermal growth factor receptor and that overexpression of this gene may be associated with tamoxifen resistance 76,77.

Because the AIs inhibit the action of aromatase, the degree of expression of this enzyme—and differences in the affinity of drug binding—could have a bearing on drug efficacy 78. Intrinsic resistance to the AIs has been suggested by variations in response rate to anastrozole and letrozole among women with ER-positive tumours 79. With respect to HER2/*neu* status and AI efficacy, neoadjuvant letrozole has been found to be equally effective in HER2/*neu*–positive and HER2/*neu*–negative tumours 80,81. However, a review of receptor status in the BIG 1-98 study, based on a relatively small number of patients with HER2/*neu*-positive tumours, showed that the prognosis of those patients was less favourable with both letrozole and tamoxifen therapy 82. Further investigations are needed to clarify the role of tamoxifen and AIs in HER2-overexpressing breast cancers, especially in the current clinical context with the adoption of adjuvant trastuzumab for these patients.

### 6.2 Tumour-Related Resistance

The estrogen receptor is a member of the nuclear receptor family of ligand-activated transcription factors. Upon entering a cell, estrogen binds to the receptor, which then undergoes conformational changes and binds to elements upstream of estrogen-dependent genes and alters their transcription either by up- or downregulation 83. When tamoxifen binds to the estrogen receptor, various conformational changes occur, and the regulation of estrogen-dependent genes changes. Thus, tamoxifen may have activity either as an agonist or an antagonist. Tamoxifen resistance may arise through changes in the expression of the receptor or through decreased uptake or increased efflux of the drug, or through changes in signalling pathways 83.

Acquired resistance to the AIs has been demonstrated in tissue-culture cell lines in which cells acquire an adaptive hypersensitivity to estrogen through upregulation of estrogen receptors and increased crosstalk between various growth factor receptor signalling pathways 78,79. Studies in a mouse tumour model have confirmed this finding. Analysis of letrozole-resistant tumours in mice revealed that the transition from a responsive state to an unresponsive state was associated with activation of growth factor receptor pathways, particularly the Her2/Raf/MAPK (mitogen-activated protein kinase) signalling pathway, and HER2 expression was increased whether the tumours were regressing or growing 84. Gene expression arrays performed on tumour biopsies in women on neoadjuvant letrozole have shown alterations in numerous genes upon exposure to letrozole, with different changes in responders and non-responders 70,85. However, the significance of this finding is not clear, and resistance likely occurs through a number of mechanisms. Cross-resistance between steroidal and nonsteroidal AIs has not been observed, as demonstrated by patients with metastatic breast cancer who responded to exemestane following failure on a nonsteroidal AI 86. Interestingly, growth of breast cancer cell lines in tissue culture is stimulated by androgens, and furthermore, under conditions of profound estrogen depletion, these cells upregulate steroidogenic enzymes that metabolize androgens to estrogen 87. This mechanism represents another potential avenue of resistance to AIs.

### 6.3 Treatment-Related Resistance

Women on tamoxifen experiencing excessive menopausal symptoms such as hot flashes may be prescribed a serotonin reuptake inhibitor (SRI), and these agents have been shown to inhibit CYP2D6 activity. Venlafaxine has been found to be a weak inhibitor of CYP2D6; paroxetine is a potent inhibitor 88. Thus, the choice of SRI for treatment is important for ensuring optimum tamoxifen activity.

## 7. SUMMARY

Data so far have unequivocally shown that, as compared with tamoxifen, the AIs offer superior benefit in DFS, and adjuvant AI treatment is accepted as the standard of care in early breast cancer. However, issues for the physician include which AI to use and when to start it. Further, questions such as duration of treatment and the optimal treatment strategy remain to be answered. The data so far point to AI treatment up front, because the risk of recurrence is highest within the first 2–3 years after surgery 1. Patients who have already been started on tamoxifen benefit from switching to an AI as demonstrated in the I TA, ARNO, and ABCSG trials, in which patients switched to anastrozole after 2–3 years of tamoxifen showed improvement in DFS. Similar results were found in the IES trial for patients switched to exemestane. A question that needs further investigation is whether, for a particular subset of patients, tamoxifen is more beneficial as initial therapy. The optimal duration for therapy is another issue that requires further investigation. The data so far indicate that extended therapy is probably effective, because letrozole and tamoxifen showed increased benefit beyond 5 years in the MA.17 and ATLAS trials respectively. In the MA.17 trial, a benefit was apparent even when substantial time had elapsed between treatment with tamoxifen and subsequent treatment with letrozole. What is unclear at present is the optimal duration of AI therapy; the SALSA trial (anastrozole) and NSABP B-42 (letrozole) will help to address that question. The ATAC 100-month analysis demonstrated a carryover effect of anastrozole that was greater than the effect known to occur with tamoxifen 15. Again, it would be useful to determine how long the carryover effect lasts and whether extended endocrine therapy will have a greater effect than that seen with the known carryover effect alone. Tissue markers are showing promise as indicators of prognosis and indicators of response. The future of the ideal endocrine treatment approach relies on further research on molecular markers and gene expression that could yield useful information to help tailor endocrine therapy for individual patients.

## Figures and Tables

**TABLE I:** Endpoints used in clinical trials for adjuvant hormone treatments for early breast cancer

*Reference*	*Investigators or study group*	*Pts* *(n)*	*Study description*	*Primary endpoints*
Fisher *et al*., 1989^6^	NSABP B14	2,644	Tamoxifen vs. placebo	OS, DFS
EBCTCG 1992^7^	EBCTCG	29,892 (40 trials)	Tamoxifen vs. no treatment meta-analysis	OS, DFS (CLBC not included within DFS definition)
EBCTCG 1998^8^	EBCTCG	36,689 (55 trials)	Tamoxifen vs. no treatment meta-analysis	OS, DFS
Baum *et al*., 2002^9^	ATAC	9,366	Tamoxifen vs. anastrozole	DFS
Goss et al., 2003^10^	MA. 17	5,187	Tamoxifen followed by letrozole (extended)	DFS
Coombes *et al*., 2004^11^	IES	4,742	Tamoxifen followed by exemestane (switch)	DFS
Boccardo *et al*., 2005^12^	ITA	448	Tamoxifen followed by anastrozole (switch)	DFS
EBCTCG 2005^1^	EBCTCG	66,000 (71 trials)	Tamoxifen vs. no treatment meta-analysis	OS, DFS
Thurlimann *et al*., 2005^13^	BIG 1–98	8,010	Tamoxifen vs. letrozole	DFS

EBCTCG = Early Breast Cancer Trialists’ Collaborative Group; OS = overall survival; DFS = disease-free survival; CLBC = contralateral breast cancer; NSABP = National Surgical Adjuvant Breast and Bowel Project; ATAC = Arimidex, Tamoxifen, Alone or in Combination; BIG = Breast International Group; IES = Intergroup Exemestane Study; ITA = Italian Tamoxifen Anastrozole trial.

**TABLE II:** Definitions of disease-free survival in breast cancer trials^14^

*Reference^a^*	*Investigators or study group*	*LR*	*DM*	*Death* *(any cause)*	*Invasive CLBC*	*Second primary*	*I/C*	*I/C LCIS*
Baum *et al.,* 2002^9^	ATAC	X	X	X	X			
Goss *et al*., 2003^10^	MA. 17	X	X		X		X	X
Coombes *et al*., 2004^11^	IES	X	X	X	X			
Jakesz *et al.*, 2005^22^	ARNO	X	X		X			
Thurlimann *et al*., 2005^13^	BIG 1–98	X	X	X	X	X		

a Subsection 1.4 provides trial details.

LR = locoregional recurrence; DM = distant metastases; CLBC = contralateral breast cancer; I/C = ipsilateral or contralateral; DCIS = ductal carcinoma *in situ*; LCIS = lobular carcinoma *in situ*; ATAC = Arimidex, Tamoxifen, Alone or in Combination; BIG = Breast International Group; IES = Intergroup Exemestane Study; ARNO = Arimidex–Nolvadex trial.

**TABLE III:** Disease-free survival in trials of aromatase inhibitors

*Strategy*	*Reference*	*Trial*	*Protocol*	*Follow-up* *(months)*	*Risk reduction (%)*	
					*Relative*	*Absolute*
Upfront	Forbes *et al*., 2008^15^	ATAC	Anastrozole vs. tamoxifen	100	15	4.1
	Mouridsen, 2008^a^	BIG 1-98	Letrozole vs. tamoxifen	76	12	2.3
Switch	Coombes *et al*., 2004^11^	IES	Tamoxifen to exemestane vs. tamoxifen	55.7	32	4.7
	Boccardo *et al*., 2006^34^	ITA	Tamoxifen to anastrozole vs. tamoxifen	64	37.5	10.5
	Jonat *et al*., 2006^35^	ABCSG 8/ARNO 95/ITA	Tamoxifen to anastrozole vs. tamoxifen	30	37.6	3.7
	Kaufmann *et al*., 2006^36^ Kaufmann *et al*., 2007^37^	ARNO 95	Tamoxifen to anastrozole vs. tamoxifen	30.1	34	4.2
Extended	Goss *et al*., 2003^10^	MA. 17	Tamoxifen to letrozole vs. tamoxifen to placebo	30	42	4.6
	Jakes *et al*., 2005^38^ Jakes *et al*., 2007^39^	ABCSG 6a	Tamoxifen to anastrozole vs. tamoxifen to placebo	62.3	38	4.7

a Mouridsen HT. Letrozole monotherapy vs. tamoxifen monotherapy or vs. letrozole in sequence with tamoxifen for post-menopausal women with endocrine-responsive early breast cancer. Presented at the San Antonio Breast Cancer Symposium 2008; San Antonio, TX; December 10–14, 2008.

ATAC = Arimidex, Tamoxifen, Alone or in Combination; BIG = Breast International Group; ITA = Italian Tamoxifen Anastrozole trial; ABCSG = Austrian Breast and Colorectal Cancer Study Group; ARNO = Arimidex–Nolvadex trial; IES = Intergroup Exemestane Study.
